# Up-Regulation of Th17 Cells May Underlie Inhibition of Treg Development Caused by Immunization with Activated Syngeneic T Cells

**DOI:** 10.1371/journal.pone.0027289

**Published:** 2011-11-08

**Authors:** Li Wang, Jinpiao Lin, Zhou Zhou, Rongfen Huo, Baihua Shen, Yue Sun, Ningli Li

**Affiliations:** 1 Shanghai Institute of Immunology, Institute of Medical Sciences, Shanghai Jiao Tong University School of Medicine, Shanghai, People's Republic of China; 2 Department of Immunology, Shanghai Jiao Tong University School of Medicine, Shanghai, People's Republic of China; Tulane University, United States of America

## Abstract

**Background:**

Our previous work showed that mice immunized with attenuated activated syngeneic T cells (aTCV) led to damping Treg function which resulted in enhancing anti-tumor immunity. It is well known that DC plays a very important role in controlling Th cell differentiation; whether DC involves Treg attenuation in immunized mice remained unknown. In this study, we provided evidence that increased mature DC (mDC) after immunization with aTCV skewed Th17 differentiation, which resulted in inhibition of Treg differentiation through IL-6 signaling pathway.

**Principal Findings:**

In the present study, we found that the frequency of mDCs increased dramatically in the immunized mice accompanied by lower Treg cells compared to the controls. Moreover, both DCs and serum derived from the immunized mice suppressed Treg differentiation *in vitro*, respectively. mDCs generated from bone marrow precursor cells *in vitro* strongly inhibited Treg development and simultaneously drove Th17 differentiation with elevated IL-6 production. However, PD-L1, a potent Treg inducer did not show effect on Treg down-regulation. Assay with transwell systems showed that cell-cell contact was necessary for IL-6 production to a threshold to activate Th17 transcriptional factor RORγt and to inhibit Treg counterpart Foxp3.

**Conclusions:**

Our results implicate up-regulated Th17 development might be one of mechanisms of enhancing anti-tumor immunity induced by immunization with aTCV, which provide a novel insight in numerous mechanisms responsible for anti-tumor immunity.

## Introduction

Treg cells have essential roles in maintenance of immune homeostasis and in regulation of effector T cell responses. Therefore, Treg cells can inhibit autoimmune reactions and impede anti-tumor immunity [1]. Depletion of Treg cells *in vivo* enhances tumor immunity in mouse models of cancer [2]. Impaired Treg function contributes to the enhanced Th1, CTL responses and thereby anti-tumor immunity [3], [4], [5], [6]. In our previous work, we found responses of anti-tumor immunity were enhanced after immunized with attenuated activated syngeneic T cells (aTCV) in the mouse model [3]. In this model, frequency and function of Treg cells were down-regulated, possibly resulted from the presence of anti-CD25 antibody, as the antibody titer was raised in mice. Serum adoptive transfer assay showed that the antibody destroyed Treg cells [4], [5]. However, there are other pathways to down-regulating Treg reported recently. Reports have shown that both Th17 and Treg differentiation depend on TGF-β signal. I*n vitro* Treg transcription factor Foxp3 was activated in the presence of TGF-β, followed by Treg development, whereas in the presence of both TGF-β and a widely expressed pro-inflammatory cytokine IL-6, Th17 transcription factor RORγt was activated which results in Th17 differentiation [7], [8]. As aTCV induces immune response of immunized mice, both IL-6 and TGF-β are existed soluble factors *in vivo*, we would like to ask whether and how Th17 differentiation was affected when Treg cells were down-regulated in aTCV immunization mice.

DCs play an important role in T cell activation and differentiation [9]. In general, the stages of DC development were delineated as 1) immature DC (imDC), showing low antigen presenting capacity with little expression of co-stimulatory and MHC II molecules; 2) mature DC (mDC), possessing the strongest antigen processing and presenting ability with high expression levels of co-stimulatory and MHC II molecules on the cell surface. It is well-known that DCs play a pivotal role in Th cells differentiation. To response to exogenous or endogenous stimulation, T cells differentiation and proliferation are skewed via interaction with DC in context of MHC II and co-stimulatory molecules [9]. In our mouse model immunized with aTCV, in which Treg cells were down-regulation accompanied with enhancing immune response, whether and how DCs involved in Treg and Th17 differentiation remained to be illustrated.

In the current study, we found that mDCs increased dramatically in aTCV immunized mice, accompanied by Treg decrease compared to those in control mice. DCs isolated from immunized mice suppressed Treg differentiation *in vitro*, which suggested that immunization stimulated DC maturation and thereby damped Treg differentiation. To analysis the mechanisms of mDC affected Treg and Th17 differentiation, we generated both imDCs and mDCs from bone marrow precursor cells and analyzed their effect on Treg and Th17 differentiation. We found that mDCs showed a strong inhibition to Treg differentiation whereas simultaneously promoting Th17 differentiation. Meanwhile, cell-cell contact was essential for the inhibition of Treg differentiation and skewing Th17 differentiation resulted from increased level of IL-6 production for activating RORγt expression and suppressing Foxp3 expression. In contrast, PD-L1 expressed on mDCs, a potential inducer of Treg, did not show effect on Treg differentiation in our model. Taken together, we provided evidence that DC maturation was necessary to promote Th17 differentiation after immunization with aTCV. Th17 generation was induced though IL-6 signaling pathway. Our results provide a novel insight in numerous mechanisms responsible for anti-tumor immunity through stimulation of Th17 differentiation *in vivo*.

## Results

### Peripheral Treg down-regulation induced by immunization with aTCV

**Figure 1:**
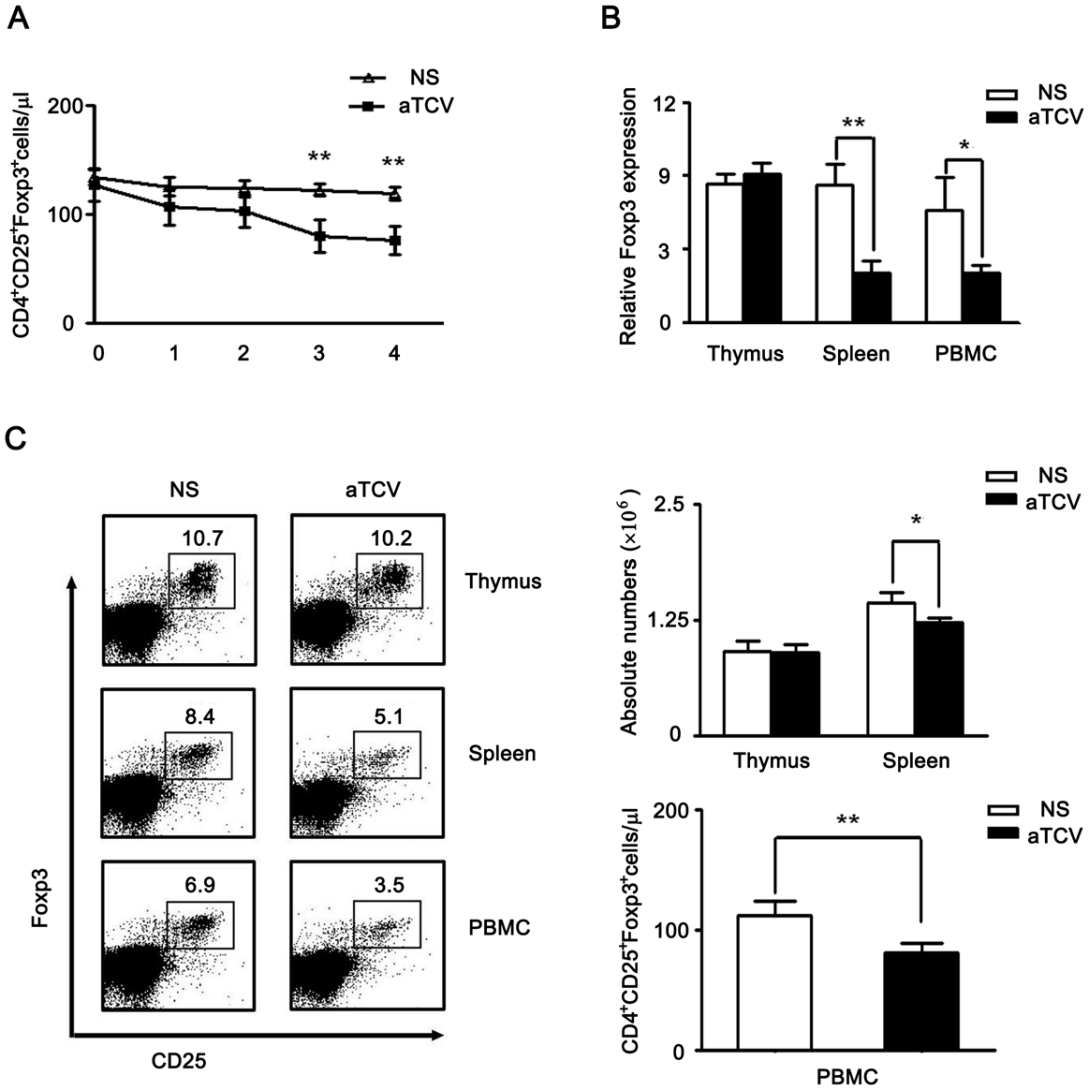
Peripheral Treg cells reduced in mice after immunization with aTCV. Naïve mice were immunized with aTCV. Mononuclear cells were isolated 5 days after immunization. A) The absolute numbers of Treg cells in peripheral blood were assessed in time course experiment. Naïve mice were immunized with aTCV at different times. Peripheral blood samples were stained with anti-CD4, anti-CD25 and anti-Foxp3 Ab. B–C, Mononuclear cells were isolated from thymus, spleen and periphery blood of mice immunized with aTCV for three times, respectively. B) The gene expression of Foxp3 was assessed by real-time PCR. C) Mononuclear cells from thymus, spleen and peripheral blood were stained with anti-CD4, anti-CD25 and anti-Foxp3 Abs and the number of Treg cells was calculated, respectively. Data are shown as mean ± SEM, n = 8–12/group, *, p<0.05, **, p<0.01.

As shown in our previous work, immunization with attenuated activated syngeneic T cells evoked anti-tumor immunity [3]. In this model, we found that the down-regulated Treg cells were involved in the anti-tumor immunity induced by immunization with aTCV [4], [5]. To assay additional mechanisms involved in the anti-immunity response, we examined immunization kinetics for Treg reduction first. Compared to the control mice, results obtained from immunized mice showed that Treg population was reduced significantly in mice received more than three times immunization (called effective immunization), whereas Treg numbers did not show differences in the mice accepted less two times immunization (Figure 1A). We also found that Treg was not changed in the mice immunized with attenuated activated B cells, attenuated resting T cells and attenuated activated allogenic T cells (data not shown). To address whether Treg cells in thymus or in the periphery were down-regulated in those received effective immunization, we examined Foxp3 gene expression in thymocytes, splenocytes and periphery blood cells (PBMC) by real-time PCR and found that the expression of Foxp3 was down-regulated in splenocytes and PBMC but not in thymocytes (Figure 1B). Consistently, CD4^+^CD25^+^Foxp3^+^ Treg population was decreased in mononuclear cell population from both PBMC and splenic cells (Figure 1C). These data indicated that in the immunized mice, Treg cells existed in periphery (consisted of induced Tregs and naturally occurring Tregs) were down-regulated but naturally occurring Treg cells in thymus did not altered. These results implied that the differentiation of Treg cells in the periphery might be reduced.

### Mature DCs induced by aTCV immunization inhibited Treg differentiation

**Figure 2:**
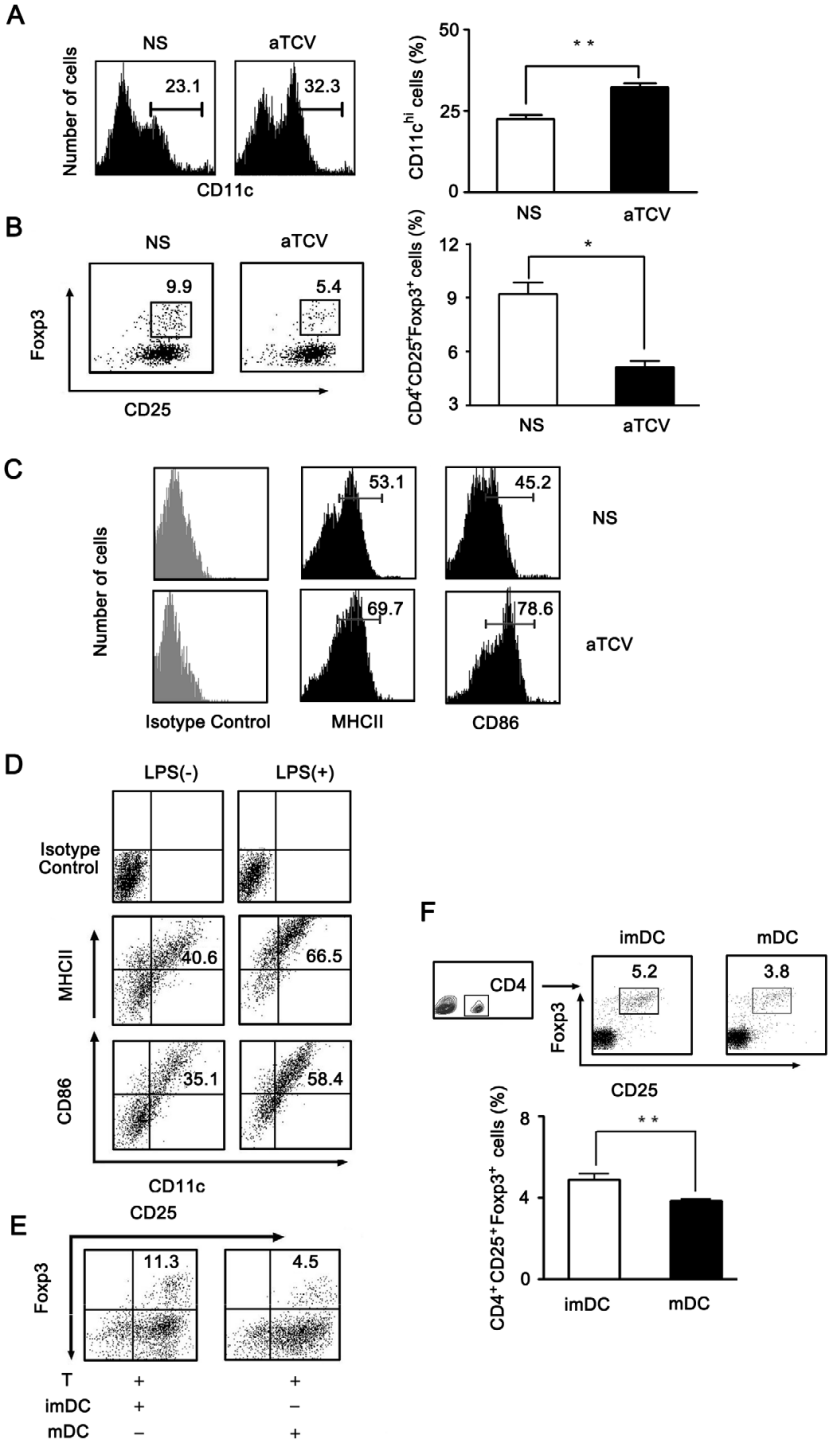
mDCs inhibited Treg differentiation. Splenocytes were isolated from mice immunized with aTCV for three times. A) Splenocytes were cultured in plates for 2 hours. The adhered cells were harvested and stained with anti-CD11c Ab. Expression of CD11c was assessed by FACS. B) DCs were purified from splenocytes using Dynabeads Mouse DC Enrichment Kit and cultured with purified CD4^+^ T cells from naïve mice. After 2-day culture, cells were stained with anti-CD4, anti-CD25, anti-Foxp3 Abs and analyzed by FACS on gated CD4^+^ T cells. C) Purified DCs were stained with anti-CD11c, anti-CD86 and anti-MHC class II Abs and analyzed by FACS on gated CD11c^hi^ cells. D–F, Bone marrow precursor cells freshly isolated from naïve mice were cultured *in vitro* in the presence of GM-CSF and IL-4. After 7 days of culture, cells were stimulated with or without LPS for 2 days. D) These cells were harvested and stained with anti-CD11c, anti-CD86 and anti-MHCII Abs. E) mDCs or imDCs generated *in vitro* were cultured with purified CD4^+^ T cells from naïve mice in the presence of TGF-β. After 2 days of culture, cells were stained with anti-CD4, anti-CD25, anti-Foxp3 Abs and analyzed by FACS on gated CD4^+^ T cells. F) mDCs or imDCs generated *in vitro* were adoptively transferred into naïve mice. After 3 days, PBMC were isolated and stained with anti-CD4, anti-CD25, anti-Foxp3 Abs. Percentage of CD4*^+^*CD25*^+^*Foxp3*^+^* cells was analyzed by FACS on gated CD4^+^ T cells. Data are shown as mean ± SEM, n = 8–12/group, *, p<0.05, **, p<0.01.

As we know DCs play a crucial role in T cell activation and differentiation. To understand whether DC was responsible for down-regulation of peripheral Treg cells in this aTCV immunization model, the changes of DCs were studied by staining with anti-CD11c–FITC. The mean fluorescent intensity of the CD11c^hi^ population was analyzed in both immunized and control mice. The percentage of CD11c^hi^ cells was higher in immunized mice than that in control mice (Figure 2A). To explore whether these altered DCs were involved in Treg aberrancy after immunization, CD4^+^ T cells from naïve mice were cultured with purified DCs from immunized mice or of control mice. The results showed that DCs from immunized mice obviously inhibited Treg differentiation compared to those from control mice (Figure 2B). Moreover, to investigate whether aTCV immunization induced DC maturation, we analyzed DCs purified from splenocytes by flow cytometry. The results showed that the percentage of CD11c^hi^CD86^+^ and CD11c^hi^MHCII^+^ cells were increased in immunized mice compared to those in control mice (Figure 2C). These data indicated that immunization with aTCV induced DC maturation, which, as a consequence, might be involved in Treg down-regulation.

To investigate the mechanisms of mDCs on Treg differentiation, DCs from bone marrow precursors in naïve mice were isolated, and then stimulated with LPS to obtain mature DCs. As reported, the expression of MHC-II and CD86 was up-regulated on CD11c^+^ cells after LPS stimulation (Figure 2D). To determine whether these mDCs could inhibit Terg differentiation, mDCs or imDCs were generated and co-cultured with CD4^+^ T cells,respectively. Results showed that only mDCs could inhibit Treg differentiation dramatically compared with those co-cultured with imDCs (Figure 2E). Furthermore, to evaluate the inhibitory effect of mDCs on Treg differentiation *in vivo*, mDCs and imDCs generated *in vitro* were adoptively transferred i.v. to syngeneic mice 3 times at 3-day interval and then Treg was analyzed after three days on the final infusion. The results showed that the CD4^+^CD25^+^Foxp3^+^ Treg population was lower in the mice received mDCs adoptive transfered (Figure 2F). These results indicated that mature DC was able to inhibit Treg differentiation *in vivo*.

### mDCs generated from bone marrow evoked Th17 differentiation

**Figure 3:**
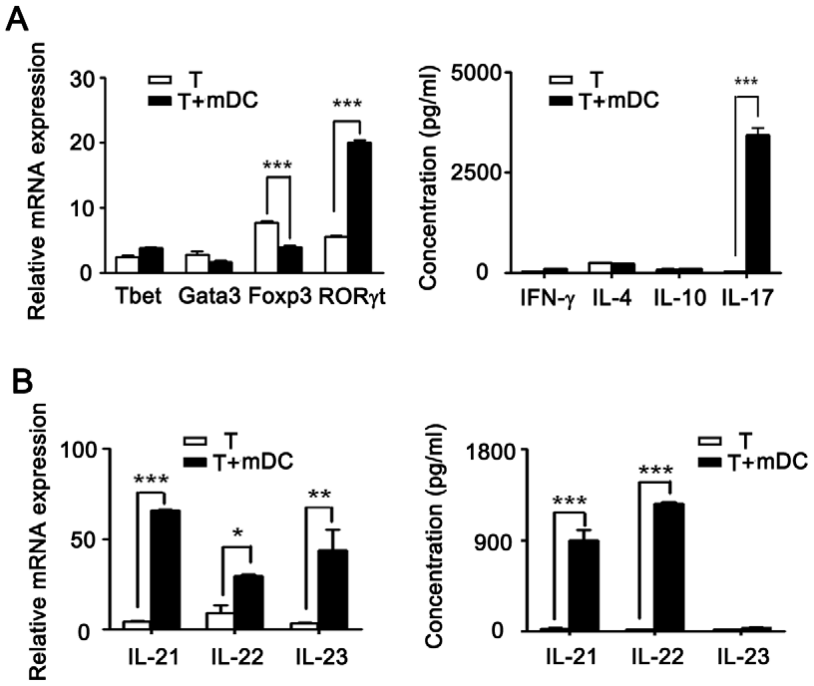
Differentiation of Th17 co-cultured with mDCs. A) Purified CD4^+^ T cells from naïve mice were cultured with mDCs generated *in vitro* in the presence of TGF-β. After culture, gene expression of T-bet, Gata3, Foxp3 and RORγt were analyzed by real-time PCR. IFN-γ, IL-4, IL-10 and IL-17 production were assessed by FlowCytomix kits. B) Gene expression and cytokine profile relative to Th17 cells were assessed. Data are representatives of three independent experiments. *, p<0.05, **, p<0.01, ***, p<0.001.

Because mDCs are well-known for guidance on Th differentiation by activation Th cells through antigen presenting, we examined T cell subsets of T cells co-cultured with mDCs. The results showed that transcription factor T-bet and Gata3 were not altered, their counterpart cytokines, IFN-γ and IL-4 were not changed. These indicated that Th1 and Th2 subsets were not affected. In contrast, the expression of RORγt was significantly increased while the expression of Foxp3 decreased simultaneously. Consistently, the level of IL-17 was significantly increased when CD4^+^ T cells co-cultured with mDCs (Figure 3A). CCR6 gene expression was also up-regulated in T cells of co-culture system (Figure S1). These data demonstrated that Th17 population increased. In line with this, both gene and protein expression of IL-21 and IL-22 were increased significantly (Figure 3B). The similar results were found in CD4^+^ T cells co-cultured with DCs from splenocytes of mice immunized with aTCV (Figure S2). These data indicated that mDCs inhibited Treg differentiation but promoted Th17 generation.

### mDCs inhibited Treg differentiation independent of PD-L1/PD-1 pathway

**Figure 4:**
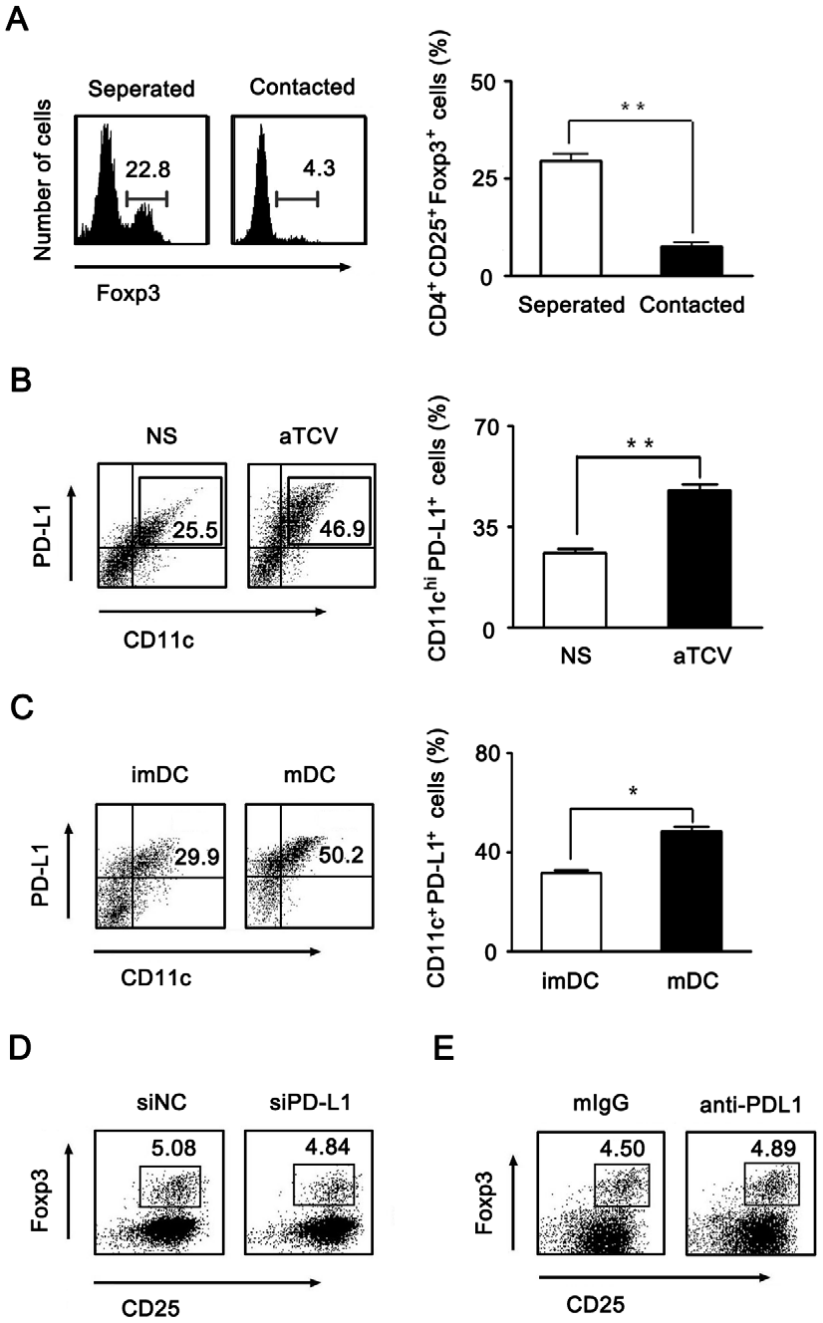
Inhibition of Treg differentiation was independent of PD-1/PD-L1 pathway. A) Purified CD4^+^ T cells were cultured with mDCs together or separately by transwell systems. Cells were harvested and stained with anti-CD4, anti-CD25 and anti-Foxp3 Abs. Foxp3 expression was assessed by FACS on gated CD4^+^CD25^+^ T cells. B-C, The frequency of CD11c^+^PDL-1^+^ cells was analyzed by FACS. B) DCs were purified from splenocytes of immunized mice using Dynabeads Mouse DC Enrichment Kit and stained with anti-CD11c and anti-PDL1 Abs. C) DCs generated from bone marrow precursor cells *in vitro* stimulated with or without LPS were stained with anti-CD11c and anti-PDL1 Ab. D) Purified CD4^+^ T cells from naïve mice were cultured with PD-L1 knockdown mDCs in the presence of TGF-β. After 2 days of culture, cells were stained with anti-CD4, anti-CD25 and anti-Foxp3 Abs and analyzed by FACS on gated CD4^+^ T cells. E) Purified CD4^+^ T cells from naïve mice were cultured with mDCs in the presence of TGF-β and anti-PD-L1 neutralizing antibody for 2 days, cells then were stained with anti-CD4, anti-CD25 and anti-Foxp3 Abs and analyzed on gated CD4^+^ T cells by FACS. Data are representatives of three independent experiments. *, p<0.05, **, p<0.01.

PD-L1 (also called B7H1) is a potential inhibitor that acts as a negative regulated co-stimulatory molecule expressed on mDCs [10]. Some studies showed that stimulation of PD-L1 molecule could induce a profound increase of CD4^+^Foxp3^+^ Treg cells differentiated from naïve CD4^+^ T cells [11]. To point out whether PD-L1 molecule involved in reduction of Treg after aTCV immunization, we investigated the inhibitory effect of mDCs on Treg differentiation using a transwell system. mDCs and CD4^+^ T cells were co-cultured but separately in a transwell system for blocking cell-cell contact. As shown in Figure 4A, the inhibited effect on Treg generation was restored without mDC contacting with CD4^+^ T cell. This indicated that cell-cell contact was essential for mDCs to execute inhibitory effect on Treg differentiation. To address whether PD-L1/PD-1 involved in the inhibitory effect, we examined and found that PD-L1 expression was high on mDCs no matter generated *in vitro* or obtained from aTCV immunized mice (Figure 4B–C). Moreover, mDCs were transfected with PD-L1 siRNA. The data showed that PD-L1 expression was 3 times lower in mDCs after siRNA transfection compared to control as shown in Figure S3. Further, mDCs generated *in vitro* or DCs isolated from mice immunized with aTCV were knocked down with PD-L1 siRNA, respectively, and then co-cultured with CD4^+^ T cells. The results showed there was no difference in Treg population when CD4^+^ T cells were co-cultured with PD-L1 knockdown mDCs or normal mDC (Figure 4D, Figure S4 ). This result provided evidence that PD-1/PD-L1 interaction did not affect inhibition of Treg differentiation mediated by mDCs (Figure 4D). Furthermore, when CD4^+^ T cells co-cultured with mDCs in the presence of neutralizing antibody of PD-L1, the Foxp3 expression was not affected also (Figure 4E). These data suggested that Treg differentiation inhibited by mDCs is independent of PD-L1/PD-1 pathway.

### Cell-cell contact played a key role in the effect of mDCs on differentiation of Treg and Th17

**Figure 5:**
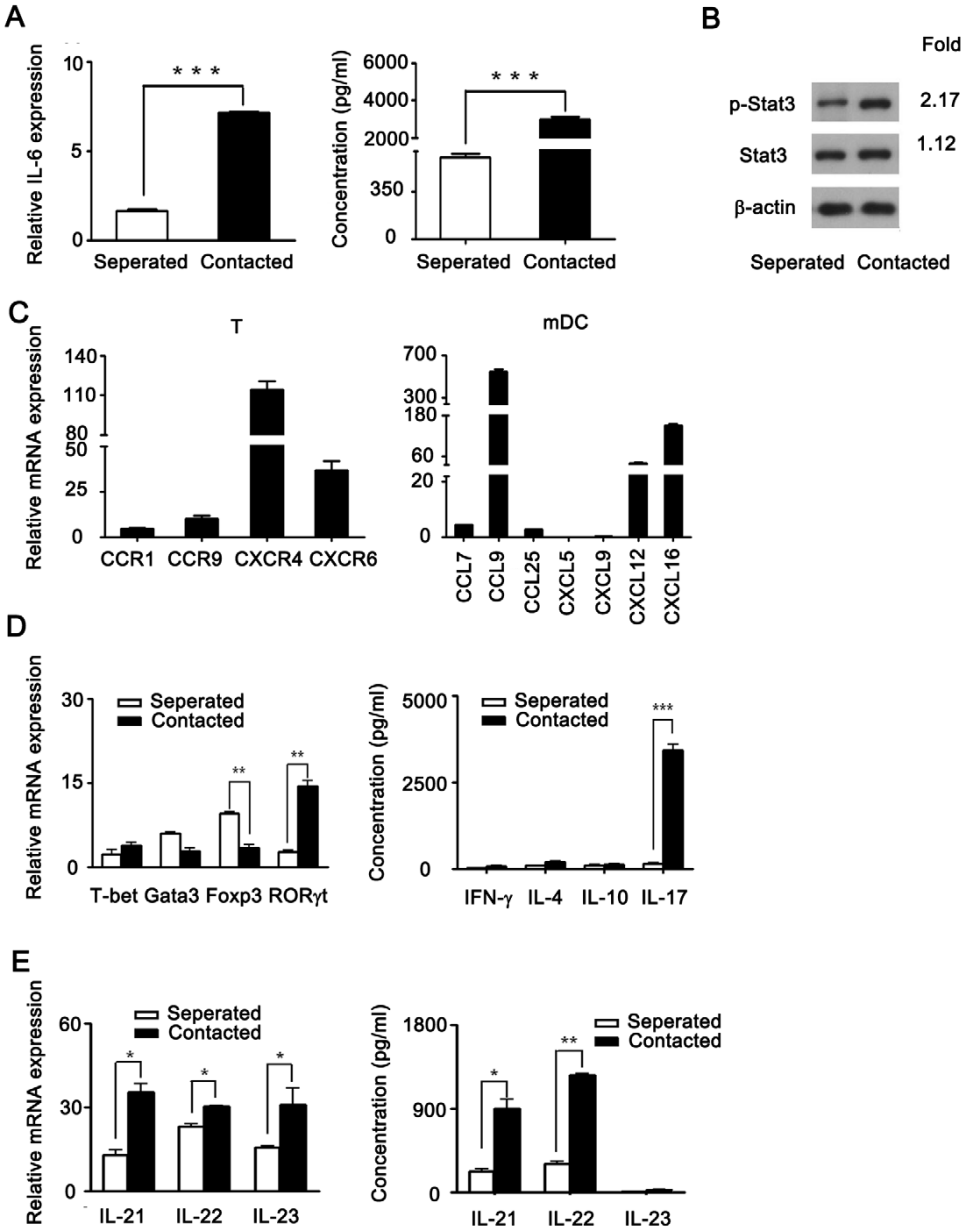
The roles of mDCs on differentiation of Treg cells and Th17 were dependent on cell-cell contact. Purified CD4^+^ T cells were cultured with mDCs together or separately by transwell systems. A) IL-6 gene expression and production of IL-6 cytokine were analyzed. B) Phosphorylation of STAT3 in total cells which included T cells and DCs was detected by Western blotting. C) Chemokine gene expression in DCs and chemokine receptor expression in T cells were analyzed by real-time PCR. D) Gene expression and cytokine production of Th1, Th2, Treg cells and Th17 were analyzed. E) Gene expression and cytokine profile relative to Th17 cells were assessed. Data are representative of three independent experiments. ***, p<0.001.

It is well known that IL-6 plays a critical role in promoting Th17 differentiation. To verify whether cell-cell contact evoked IL-6 production, we investigated IL-6 production. The results showed that the expression of IL-6 gene and protein was much higher in mDCs co-cultured with CD4^+^ T cells compared with those cultured separately (Figure 5A), meanwhile, RORγt expression increased compared with Foxp3 decreased. Moreover, when mDCs co-cultured with CD4^+^ T cells in the presence of anti IL-6 neutralizing Ab, the expression of Foxp3 was increased while RORγt decreased (Figure S5). These results indicated that mDCs promoted Th17 differentiation, and this effect might go through the IL-6 signaling pathway as STAT3 was phosphorylated significantly (Figure 5B). To investigate how mDCs interacted with T cells, expression levels of chemokines and chemokine receptors on CD4^+^ T cells and mDCs were examined. Results showed that CD4^+^ T cells mainly expressed CXCR4 and CXCR6, whereas mDCs expressed CCL7, CCL9, CCL25, CXCL12 and CXCL16 (Figure 5C). These data indicated that mDCs secreted chemokines (for example, CXCL12 and CXCL16) to recruit CD4^+^ T cells expressed CXCR4 and CXCR6, which led to mDC and CD4^+^ T cells contacting and evoked IL-6 production.

In order to understand whether the level of IL-6 increased contributed to Th17 differentiation, the expression levels of genes related to differentiation of Th1, Th2, Treg and Th17 cells were assessed by real-time PCR. The data showed that when mDCs were separated with CD4^+^ T cells by a transwell system, expression of Foxp3 was increased, the expression of RORγt was decreased, consistently with IL-17 production decreased (Figure 5D). Moreover, the gene expression levels and protein levels of IL-21 and IL-22 were decreased simultaneously (Figure 5E). Taken together, these results suggested that cell-cell contact is necessary for mDCs effect on Treg and Th17 differentiation.

### Th17 differentiation evoked by mDCs inducing increased secretion of IL-6

**Figure 6:**
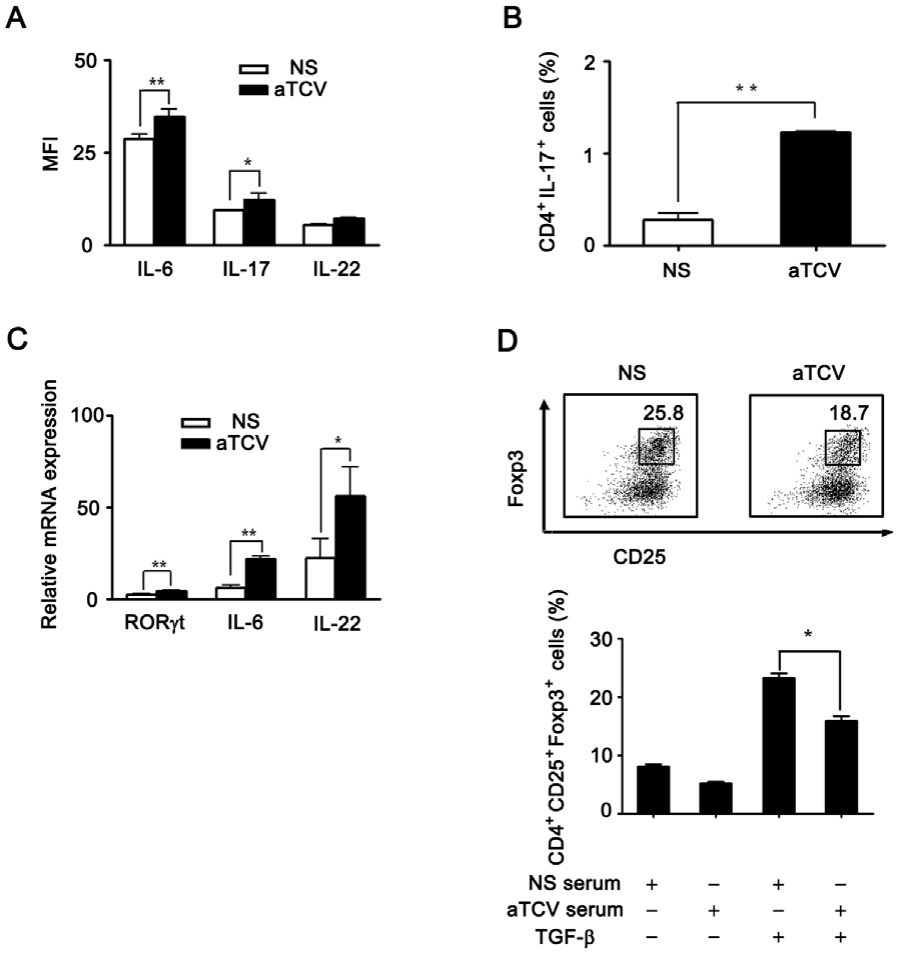
aTCV promoted differentiation of Th17 *in vivo*. Naïve mice were immunized with aTCV for three times. Five days after final immunization, sera and splenocytes were isolated. A) The levels of cytokine production were measured by FlowCytomix kits as indicated. B) Splenocytes were restimulated with PMA/ionomycin for 5 h and intracellular IL-17 production was measured by flow cytometry. C) Gene expression was assessed as indicated by real-time PCR. D) Purified CD4^+^ T cells from naïve mice were cultured with 2% serum from aTCV immunized or control mice with or without TGF-β. After 2-day culture, cells were stained with anti-CD4, anti-CD25, anti-Foxp3 Abs and analyzed by FACS on gated CD4*^+^* T cells. Data are representatives of three independent experiments. *, p<0.05, **, p<0.01.

To evaluate whether the level of IL-6 was changed in the mice immunized with aTCV, cytokine profiles in serum were assessed. As shown in Figure 6A, the levels of IL-6 and IL-17 were increased in sera isolated from the mice immunized with aTCV compared with the control mice. Compatibly, percentage of Th17 in CD4^+^ T cell population was increased significantly (Figure 6B). The gene expression of IL-6, RORγt and IL-22 were also increased (Figure 6C). To verify the sera from immunized mice inhibited Treg differentiation, CD4*^+^* T cells cultured with immunized mouse serum in the presence of TGF-β. The data showed that sera from immunized mice conveyed significant inhibition roles on Treg differentiation and increase Th17 differentiation (Figure 6D). These results suggested that increased mDCs, which promoted IL-6 secretion, induced Th17 differentiation and inhibited Treg generation *in vivo*.

## Discussion

Treg cells represent a substantial obstacle for development of cancer immunotherapy. Numerous strategies were under active investigation to achieve Treg depletion *in vivo*, including low dose cyclophosphamide, interleukin-2 immunotoxins or CD25 antibody treatment [12], [13], [14], [15]. There were very few studies through syngeneic cell immunization to reduce Treg cells until we reported our studies in 2006 [3].

In our previous studies, we found that immunization with aTCV enhanced Th1 and CTL activity, leading to augmentation of anti-tumor immunity accompanied by damped Treg function [5]. In this study, we confirmed that Treg population significantly reduced only in the mice immunized with aTCV, with no difference of Treg numbers in the mice immunized with attenuated activated B cells, attenuated resting T cells and attenuated activated allogenic T cells (data not shown). As syngeneic T cells are different from allogenic T cells regarding to their MHC molecules, syngeneic aTCV could down-regulate Treg cells, whereas allogenic aTCV could not, suggesting that MHC molecules were not involved in the Treg reduction in this model. In the present study, we found that immunization with aTCV did not affect nTreg development in thymus. However, Treg cells in periphery were decreased, indicating that Treg reduction might be to some extent due to the generation of decreased Treg cells in periphery.

DCs play a very important role in altering T cell differentiation upon cell-cell contact. By immunization, DCs underwent a maturation process resulted in morphological and functional changes by which DCs were divided into two conventional subsets: imDCs and mDCs [16], [17]. Among them, which population is responsible for Treg down-regulation is unclear. In this study, we showed that mDCs, which increased in spleen of aTCV immunized mice, could significantly inhibit Treg differentiation but promote Th17 development, as well as those of cultured from bone marrow precursors *in vitro*, suggesting that DC maturation had impact on Treg and Th17 differentiation. Our data were consistent with that described previously [18], [19], [20], these authors found that Treg cells could effectively suppress CD4^+^CD25^−^ T cell activation in a co-culturing system where freshly isolated splenic DCs were used as APCs, while stimulation of DCs with LPS reversed Treg-mediated suppression and restored T cell proliferation. In these reports, IL-6 secreted by DCs was required for overcoming suppression. Our data supported that maturation of DCs affected Treg differentiation also. While the mechanisms of DC maturation in aTCV immunized mice remains unknown and more extended study to identify how the DC maturation induced by aTCV immunization *in vivo* is under investigation.

DCs stimulated with LPS could induce IL-6 production [21]. IL-6 promotes the generation of Th17 cells from naïve T cells together with TGF-β. As immunization with aTCV initiated DC maturation, IL-6 increased *in vivo* which was consistent with the up-regulation of RORγt, IL-17 and IL-22. We also found that CXCL12 and CXCL16 on mDCs were highly expressed. These results implied that mDCs through up-regulation of chemokines recruited CXCR4 and CXCR6 of CD4^+^ T cells [22], [23], [24] and then, differentiated T cells to Th17. In our study, Th2 subset did not alter obviously. It is consistent with the report that TGF-β is found to interfere with Th2 differentiation *in vitro* [25]. Although we found that Th1 increased after immunization with aTCV [4], but further study *in vitro* did not found the increased expression of T-bet and IFN-γ. The reason might be that existed TGF-β, a critical antagonist for Th1 differentiation, affects expression of IFN-γ and T-bet [26], [27]. Our study supported and was consistent with previous reports which demonstrated that TGF-β blocked Th1 and Th2 differentiation but promoted Th17 differentiation simultaneously [28]. This is very important to add knowledge about mDC involved in Th differentiation in immune response. IL-10 is a cytokine with anti-inflammatory properties and secreted by several immune cells [29]. In our experiment system, we did not detected high levels of IL-10 production.

PD-L1, also called B7H1, as a co-stimulatory molecule expressed on mDCs is a potential inhibitor in immune response [10]. It was reported that blockade of an interaction between PD-L1 and PD-1 would result in aberration on expansion and function of Treg cells [11]. However, some studies showed that stimulation of PD-L1 molecule could induce a profound increase of CD4^+^Foxp3^+^ Treg cells differentiated from naïve CD4^+^ T cells [30]. As we found that mDCs involved in impaired Treg differentiation, we hypothesized that silencing PD-L1 would restore Treg differentiation. Unexpectedly, the results showed neither silencing PD-L1 expression by RNAi, nor blocking PD-L1 with neutralizing antibody, could restore inhibition of Treg differentiation induced by mDCs. These data suggested that PD-1/PD-L1 pathway is not involved in the process. However, this result implied strongly that some soluble factors derived from mDCs are accountable for Treg reduction. By using the transwell system, we confirmed that mDC released IL-6 to prompt Th17 differentiation and inhibit Treg differentiation simultaneously. These were supported by elevated IL-6, IL-17, IL-21 and IL-22 production *in vivo* and *in vitro*. In another word, mDCs are very important to skew CD4^+^ T cells into Th17 but inhibit Treg differentiation. We identified that only mDC could promote Th17 differentiation in receipt mice by adoptive transfer assay. However, in the current study, we failed to detect increased IL-23 production both *in vitro* and *in vivo*. As a member of the IL-12 family, IL-23 is not involved in the initial differentiation but might serve to expand and stabilize Th17 responses [31]. How Th17 is expanded and stabilized in the aTCV immunized mouse model are now being investigated.

Why separation of mDCs and CD4^+^ T cells with the transwell system blocked Th17 differentiation and meanwhile rescued Treg differentiation? By analysis chemokine profile, we found that, although expression levels of CXCL12 and CXCL16 on mDCs were not changed (data not shown), the transwell membrane blocked cell-cell contact of mDC and CD4^+^ T cell which was necessary for IL-6 production. From these data, we concluded that mDCs contacted with CD4^+^ T cells resulted in IL-6 production increased. Without IL-6, not Th17 but Treg cells were differentiated in the presence of TGF-β. This suggested that cell-cell contact is necessary for polarizing cytokine IL-6 production. This is very important to propose that aTCV immunization could evoke a seesawing effect *in vivo*: up-regulation of Th17 differentiation and down-regulation of Treg differentiation, which led to enhancing immune response and further arresting tumor growth *in vivo* [3]–[5].

In recent years, the function of Th17 cells and IL-17 is unclear in anti-tumor immunity [32]. Several reports observed that Th17 cells and IL-17 had been found in various human tumors [33], [34], [35], [36]. In contrast, the expression of IL-17 in a hematopoietic origin tumor was reported to promote protection in immunocompetent hosts including to rise tumor-specific CD8^+^ T cell [37], [38], [39], [40]. Therefore, immunotherapy targeting on enhancing Th17 activity might benefit cancer patients. Our study offers a model to understand how to enhance Th17 function via simultaneously inhibiting Treg *in vivo*.

**Figure 7:**
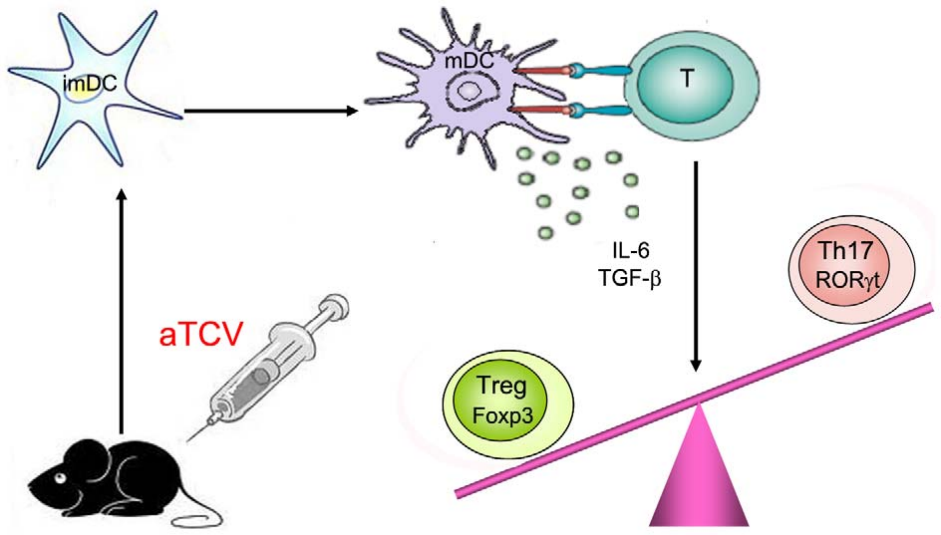
Schematic model of Th17 differentiation with aTCV immunization. After aTCV immunization, increased mDCs interacted to T cells, which induced IL-6 production and Th17 differentiation followed by inhibition of Treg generation.

Taken together, our results implicated that DC maturation played an important role in promoting Th17 differentiation and inhibiting Treg generation. After aTCV immunization, the expression of RORγt and the protein levels of IL-6, IL-17 increased whereas Foxp3 expression decreased *in vivo* (Figure 7). Based on our finding that aTCV could enhance DC maturation, we suggest that up-regulated Th17 development might be one of mechanisms of enhancing anti-tumor immunity induced by immunization with aTCV, which provide a novel insight in numerous mechanisms responsible for anti-tumor immunity. Given these considerations, more extended study to identify how the DC maturation induced by aTCV immunization *in vivo* is under investigation.

## Materials and Methods

### Ethical treatment of animals

This study was carried out in strict accordance with the recommendations in the Guide for the Animal Care and User Committee guidelines. The protocol was approved by the Committee on the Ethics of Animal Experiments of Shanghai Jiao Tong University School of Medicine (Permit Number: 2010039).

### Animals

6–8 week-old female C57BL/6 mice were purchased from Shanghai Slac Laboratory Animal Co., and were kept under pathogen-free conditions at the animal core facility of Shanghai Jiao Tong University School of Medicine.

### Attenuated activated syngeneic T cell preparation and administration

Attenuated activated syngeneic T cells were prepared and administrated for immunization as described in our previous report. Briefly, Splenocytes from naïve mice were stimulated with 5 µg/ml ConA (Vector Laboratories, Burlingama, CA, USA) in complete DMEM (DMEM with 10% fetal calf serum, 10 mM HEPES, 50 µM β-mercaptoethanol, 2 mM L-glutamine and 50 IU/ml penicillin-streptomycin) in 25 ml flasks for 72 h. The cells were collected and washed three times with PBS (pH 7.4). T cells were then purified using Dynabeads coated with specific antibodies by negative selection (Dynal Biotech, Oslo, Norway). The purity of T cells obtained from culture was greater than 95%. Purified T cells were irradiated at 3000 rad. Mice were immunized by intraperitoneal and subcutaneous injection with 5×10^6^ cells/mouse. The mice received similar immunizations every 5 days. Five days after final immunization, the mice were killed.

### CD4^+^ T cell isolation

Spleens from naïve mice were freshly removed and prepared for single-cell suspensions. CD4^+^ T cells were negatively isolated from the resulting splenocytes using magnetic bead separation. Briefly, splenocytes were depleted for CD8^+^, B220^+^, CD16^+^, Gr-1^+^ and Ly76^+^ cells using biotin-labeled specific mAb (BD Biosciences PharMingen, San Diego, CA, USA), anti-biotin magnetic beads, and an LD magnetic bead column (Miltenyi Biotec, Auburn, CA, USA). The purity of CD4^+^ T cells was always greater than 95%, as indicated by flow cytometry.

### Dendritic cell from spleen isolation

Fresh spleens were removed and prepared for single-cell suspensions. DCs were purified (≥93%) using Dynabeads Mouse DC Enrichment Kit (Invitrogen Dynal AS, Oslo, Norway) according to the manufacturer's instructions.

### Dendritic cell Generation

Bone marrow-derived dendritic cells were prepared using methods as described by Inaba et al. (1992). Briefly, bone marrow cells were flushed from the femurs of naïve C57BL/6 mice using complete RPMI 1640 (cRPMI) medium (RPMI 1640 medium (Sigma, Oakville, ON) supplemented with 10% fetal bovine serum (FBS, Gibco, Burlington, ON), 100 U/ml penicillin, 100 µg/ml streptomycin, 2 mM glutamine and 2-mercaptoethanol). Red blood cells were lysed by adding 1 ml ACK buffer (Lonza, Walkersville, MD). The bone marrow cells were washed twice with cRPMI and plated at a density of 2×10^6^ cells/ml in 6-well plates. Cells were cultured in fresh DC culture medium (cRPMI media with 10 ng/ml GM-CSF and 1 ng/ml IL-4 (PeproTech, Rocky Hill, NJ). On days 3 and 5, non-adherent cells were washed away, and fresh DC culture medium was added to the culture plate at these time points. On day 7, 100 ng/ml LPS (Sigma) were added to stimulate DCs to mature. On day 9 BMDCs were purified (≥93%) using Dynabeads Mouse DC Enrichment Kit (Invitrogen Dynal AS, Oslo, Norway) according to the manufacturer's instructions.

### DC with CD4^+^ T cell co-culture

Purified CD4^+^ T cells from naïve mice were resuspended at the concentration of 1×10^6^/ml in cRPMI and cultured with fresh purified DCs from spleens of mice or from cultured DCs *in vitro* at the ratio of 5∶1 in the presence of 2 µg/ml of platebound anti-CD3 (clone 145-2C11, eBioscience), 2 ug/ml soluble anti-CD28 (clone 37.51, eBioscience), 2 ng/ml TGF-β (R&D Systems) and 100 U/ml IL-2 (R&D Systems). After 24 hr, the cells were harvested and gene expression was analyzed by real-time PCR. After 48 hr-culture, the cells were harvested and stained with anti-CD4, anti-CD25 and anti-Foxp3 Ab. Foxp3 expression were assessed by FACS. A transwell system (0.4 µm pore, Costar, Corning Inc., Corning, NY, USA) was used in the co-culture. Purified CD4^+^ T cells were seeded in the apical chamber of transwell with 0.4 µm pore. DCs were cultured in the lower compartment of the transwell. The cells were maintained in a 37°C 5% CO2 incubator. After 24 hr or 48 hr of culture, gene expression, cell surface markers and cytokine production were analyzed.

### siRNA oligo transfection

PD-L1 small interfering RNA (siRNA) was designed and synthesized at Shanghai GenePharma (Shanghai, China). mDCs were precoated on 12-well plates (Costar, Corning Incorporated). The PD-L1 siRNA oligo and the GenePORTER reagent (Gene Therapy Systems) were diluted with serum-free medium, then mixed and incubated at room temperature for 20 min. Culture medium was aspirated from the cells, the mixture added to the cells and the culture incubated at 37°C for 3–5 h. Post-transfection, 1 volume of medium containing 20% FCS was added and the culture incubated overnight under 5–10% CO_2_ at 37°C. After 24 hr, addition of purified CD4^+^ T cells was added in the presence of 2 ug/ml of anti-CD3(clone 145-2C11, eBioscience), 2 ug/ml soluble anti-CD28 (clone 37.51, eBioscience), 2 ng/ml TGF-β (R&D Systems) and 100 U/ml IL-2 (R&D Systems) for 2days. After this period of culture, cell surface markers were assessed.

### Flow Cytometry analysis

Cells were washed with PBS and then stained with various fluorochromes using standard methods provided by the manufacturers. Antibodies used for surface staining of FITC labeled anti-CD4, -CD11c, PE labeled anti-CD86, -MHCII, -PD-L1 and APC labeled anti-CD25 Abs were purchased from BD PharMingen. For detection of Foxp3^+^ cells or IL-17^+^ cells, these cells were fixed and permeabilized according to the manufacturer's instructions and incubated with PE labeled anti-Foxp3 mAb, PE labeled anti- IL-17 mAb (e-Bioscience, San Diego, CA, USA), respectively. Isotype-matched antibodies were used as controls, and cells were preincubated with mouse IgG to avoid nonspecific binding to FcRs. All samples were analyzed using a BD FACSCalibur flow cytometer (Becton Dickinson, San Jose, CA, USA).

### Cytokine measurement

FlowCytomix kits which measure cytokines IL-6, IL-10, IL-17, IL-22, IL-23, IFN-γ and IL-4 were purchased from Bender MedSystems (Vienna, Austria). The levels of IL-21 were measured by ELISA. The measurement of cytokines was performed according to the manufacturer's instructions. Standard curves for each cytokine were generated by using the reference cytokine concentrations supplied by the manufacturers. Raw data of the FC bead assay were analyzed by FlowCytomixPro1.0 software.

### RNA extraction and gene expression analysis

RNA extraction and real-time PCR was performed as previously reported [3]. The primers used in this study were showed in table 1.

**Table 1:** Specific primers used in real-time PCR analysis.

Gene	Prime: Fw: 5′-3′	Prime: Rv: 5′-3′
IL-6	TTCCATCCAGTTGCCTTCTTG	GGGAGTGGTATCCTCTGTGAAGTC
IL-21	GCATGCAGCTTTTGCCTGTT	CACGAGGTCAATGATGAATGTCTTA
IL-22	CAACACCCGGTGCAAGCT	TCCTTGGCCAGCATAAAGGT
IL-23	TGGCTGTGCCTAGGAGTAGCA	GCATGCAGAGATTCCGAGAGA
RORγt	CACGGCCCTGGTTCTCAT	GCAGATGTTCCACTCTCCTCTTCT
Foxp3	AGGAGCCGCAAGCTAAAAGC	TGCCTTCGTGCCCACTGT
T-bet	ATGTTTGTGGATGTGGTCTTGGT	CGGTTCCCTGGCATGCT
Gata3	CAAGCTTCATAATACCCCTGACTATG	GCGCGTCATGCACCTTTT
CCR1	GCAGAGAGGAAAGACAGAACACTTT	TCTTTTTTATGGTTGGCTGCCTAT
CCR9	TAGTTTGGAGCCACCTGTCAGA	GAAGTGGATTGGGAGCAAGACT
CXCR4	CTTTGTCATCACACTCCCCTT	GCCCACATAGACTGCCTTTTC
CXCR6	CCAAGTCATTTGCTTGCTCATTT	TCAATATCTTGAACATGGCCATAGA
CCL7	GGGTCGAGGAGGCTATAGCAT	TTCTGTTCAGGCACATTTCTTCA
CCL9	CAACAGAGACAAAAGAAGTCCAGAG	CTTGCTGATAAAGATGATGCCC
CCL25	TTACCAGCACAGGATCAAATGG	GGTTGCAGCTTCCACTCACTT
CXCL5	TCCCCAGCGGTTCCATCT	CGTGAACAGCAACAGAAATGC
CXCL9	TGGAGCAGTGTGGAGTTCGA	CCTCGGCTGGTGCTGATG
CXCL12	AGAGCCAACGTCAAGCATCTG	TCTTCAGCCGTGCAACAATC
CXCL16	GCAGTGTCGCTGGAAGTTGTT	GATCCAAAGTACCCTGCGGTATC
β-actin	TGTCCA CCT TCC AGC AGA TGT	AGC TCA GTA ACA GT C CGC CTA G

### Western blot analysis

Cells were harvested and lysed with 50 mM Tris–HCl buffer (pH 7.4) containing 150 mM NaCl, 5 mM EDTA, 1% Nonidet P-40, 0.5% sodium deoxycholate, 0.1% SDS, and protease inhibitor mixture (Roche Applied Science). For each sample, equal amounts of 30 µg protein were electrophoresed and transferred to Immobilon-P membranes (Millipore, Bedford, MA). Membranes were blocked in 5% nonfat milk and 0.1% Tween 20 in PBS, and probed with primary antibodies of STAT3, phospho-STAT3 and β-actin (Cell Signaling Technology) diluted in 1% nonfat milk and 0.1% Tween 20 in PBS. Membranes were washed 3 times with PBS-Tween (PBST), and probed with donkey anti-goat horseradish peroxidase-conjugated secondary antibodies (Santa Cruz Biotechnology) in 1% nonfat milk in PBST. Membranes were washed 3 times for 20 min with PBST. The enhanced chemiluminescence (ECL) system (Amersham Pharmacia Biotech, Piscataway, NJ) was used to visualize bands.

### Adoptive transfer

2×10^6^ DCs were injected into naïve mouse by intravenous injection (i.v.). The CD4^+^CD25^+^Foxp3^+^ T cells in peripheral blood mononuclear cells (PBMC) of recipient mice were analyzed by FACS after 3 days.

### Statistical analysis

Except where indicated otherwise, data are expressed as the mean±SEM. Student's t-test was used to analyze the differences between groups. Statistically significant changes were first determined by one-way analysis of variance and then by Student's paired or unpaired 2-tailed.

## Supporting Information

Figure S1 **CCR6 gene expression on T cells.** Purified CD4^+^ T cells from naïve mice were cultured with or without mDCs generated *in vitro* in the presence of TGF-β. After culture, CCR6 gene expression was assessed in purified T cells by real-time PCR. Data are representatives of three independent experiments. *, p<0.05.
(TIF)

Figure S2 **Differentiation of Th17 co-cultured with DCs from splenocytes of immunized mice.** A) Purified CD4^+^ T cells from naïve mice were cultured with DCs isolated from splenocytes of immunized mice in the presence of TGF-β. After culture, gene expression of T-bet, Gata3, Foxp3 and RORγt were analyzed by real-time PCR. IFN-γ, IL-4, IL-10 and IL-17 production were assessed by FlowCytomix kits. B) Gene expression and cytokine profile relative to Th17 cells were assessed. Data are representatives of three independent experiments. *, p<0.05, **, p<0.01.
(TIF)

Figure S3 **siRNA suppressed PD-L1 expression.** mDCs generated in vitro were knocked down by siPD-L1. PD-L1 gene expression was assessed by real-time PCR. Data are representatives of three independent experiments. ***, p<0.001.
(TIF)

Figure S4 **PD-L1 Knockdown DCs from immunized mice did not restore Treg differentiation.** Purified CD4^+^ T cells from naïve mice were cultured with PD-L1 knockdown DCs from splenocytes of mice immunized with aTCV in the presence of TGF-β. After 2 days of culture, cells were stained with anti-CD4, anti-CD25 and anti-Foxp3 Abs and analyzed by FACS on gated CD4^+^ T cells. Data are representatives of three independent experiments.
(TIF)

Figure S5 **Inhibition of Treg differentiation was dependent on IL-6.** Purified CD4^+^ T cells from naïve mice were cultured with mDCs in the presence of TGF-β and anti-IL-6 neutralizing antibody for 2 days, cells then were stained with anti-CD4, anti-CD25 and anti-Foxp3 Abs and analyzed on gated CD4^+^ T cells by FACS. Data are representatives of three independent experiments. **, p<0.01.
(TIF)
