# Supplementary figures and images for: Up-Regulation of Th17 Cells May Underlie Inhibition of Treg Development Caused by Immunization with Activated Syngeneic T Cells
Source: PLoS One. 2011 Nov 8;6(11):e27289. doi: 10.1371/journal.pone.0027289 (PMC3210778; doi:10.1371/journal.pone.0027289)

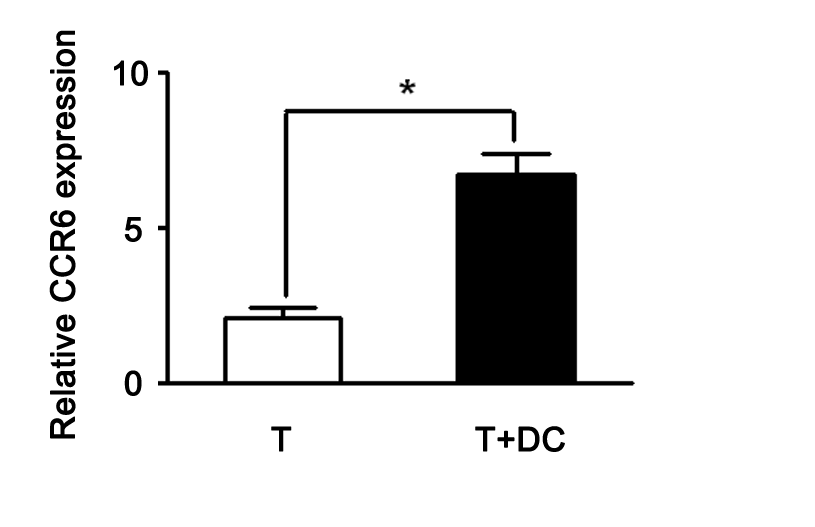

Supplement: Figure S1 — CCR6 gene expression on T cells. Purified CD4+ T cells from naïve mice were cultured with or without mDCs generated in vitro in the presence of TGF-β. After culture, CCR6 gene expression was assessed in purified T cells by real-time PCR. Data are representatives of three independent experiments. *, p<0.05. (TIF) [file pone.0027289.s001.tif]

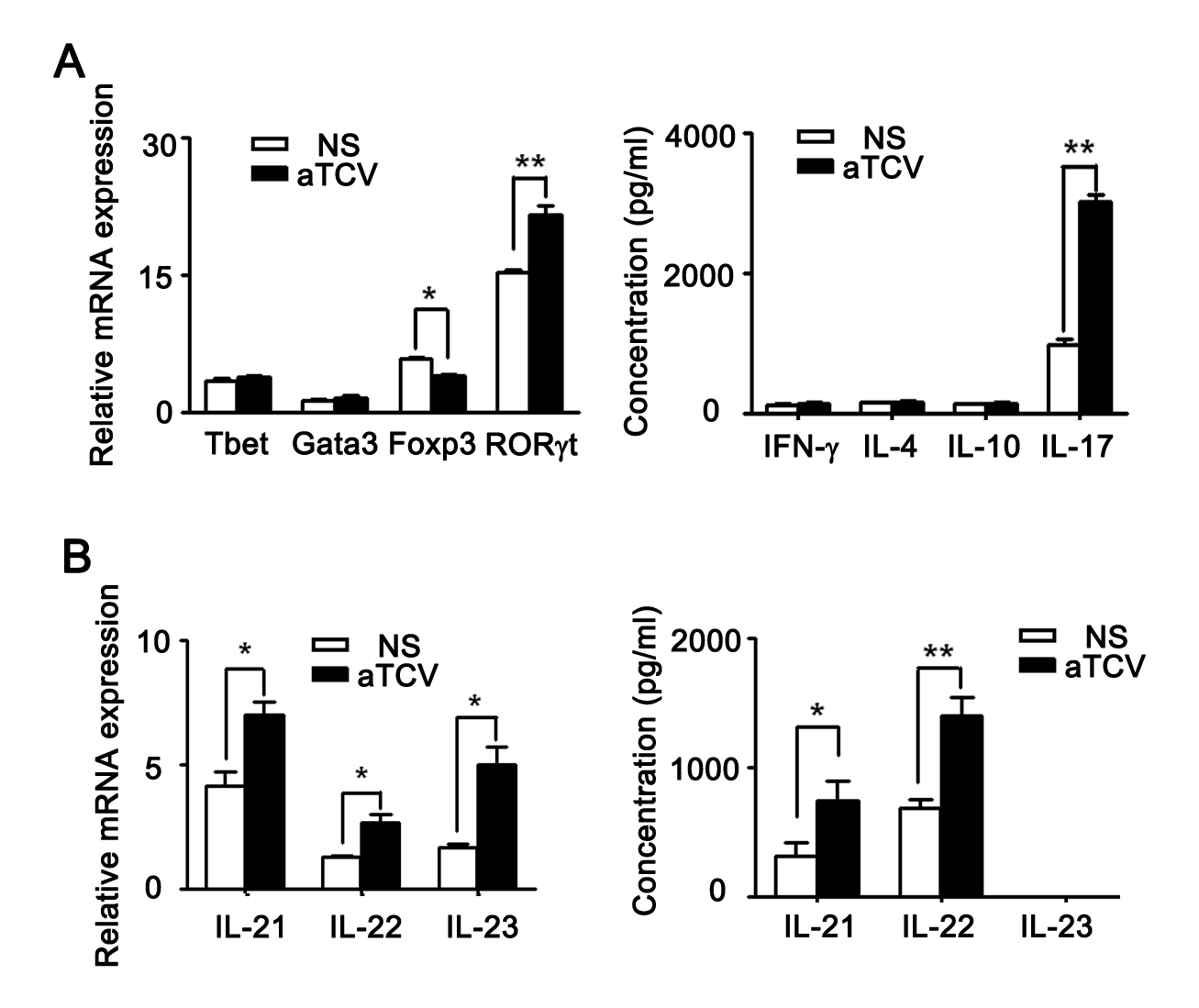

Supplement: Figure S2 — Differentiation of Th17 co-cultured with DCs from splenocytes of immunized mice. A) Purified CD4+ T cells from naïve mice were cultured with DCs isolated from splenocytes of immunized mice in the presence of TGF-β. After culture, gene expression of T-bet, Gata3, Foxp3 and RORγt were analyzed by real-time PCR. IFN-γ, IL-4, IL-10 and IL-17 production were assessed by FlowCytomix kits. B) Gene expression and cytokine profile relative to Th17 cells were assessed. Data are representatives of three independent experiments. *, p<0.05, **, p<0.01. (TIF) [file pone.0027289.s002.tif]

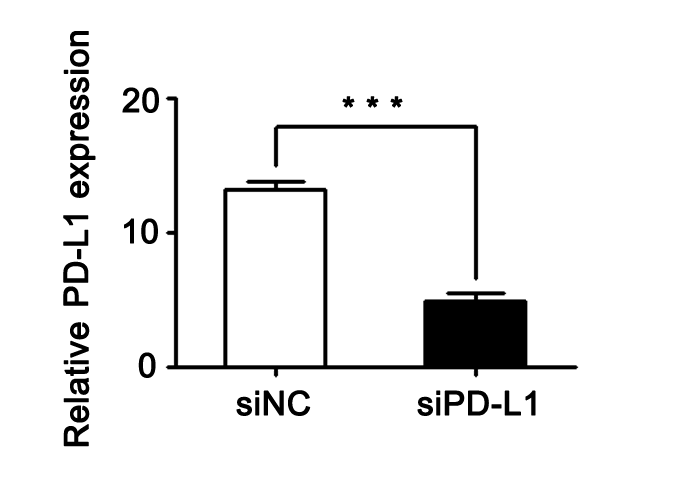

Supplement: Figure S3 — siRNA suppressed PD-L1 expression. mDCs generated in vitro were knocked down by siPD-L1. PD-L1 gene expression was assessed by real-time PCR. Data are representatives of three independent experiments. ***, p<0.001. (TIF) [file pone.0027289.s003.tif]

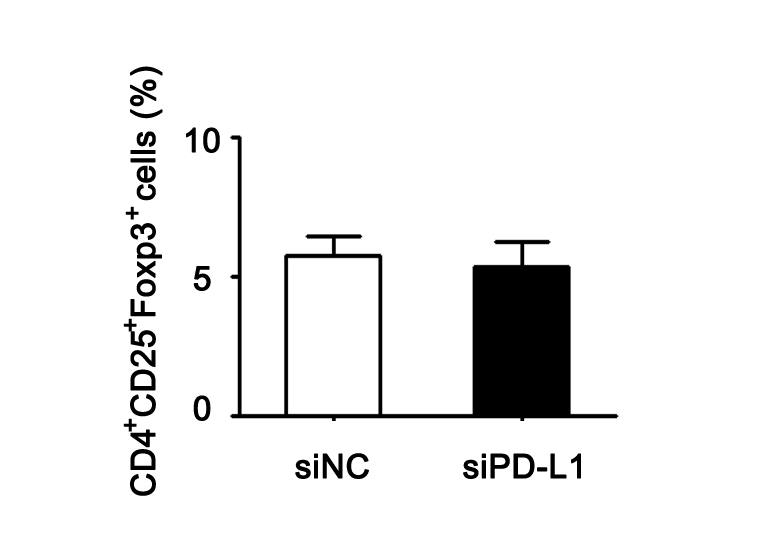

Supplement: Figure S4 — PD-L1 Knockdown DCs from immunized mice did not restore Treg differentiation. Purified CD4+ T cells from naïve mice were cultured with PD-L1 knockdown DCs from splenocytes of mice immunized with aTCV in the presence of TGF-β. After 2 days of culture, cells were stained with anti-CD4, anti-CD25 and anti-Foxp3 Abs and analyzed by FACS on gated CD4+ T cells. Data are representatives of three independent experiments. (TIF) [file pone.0027289.s004.tif]

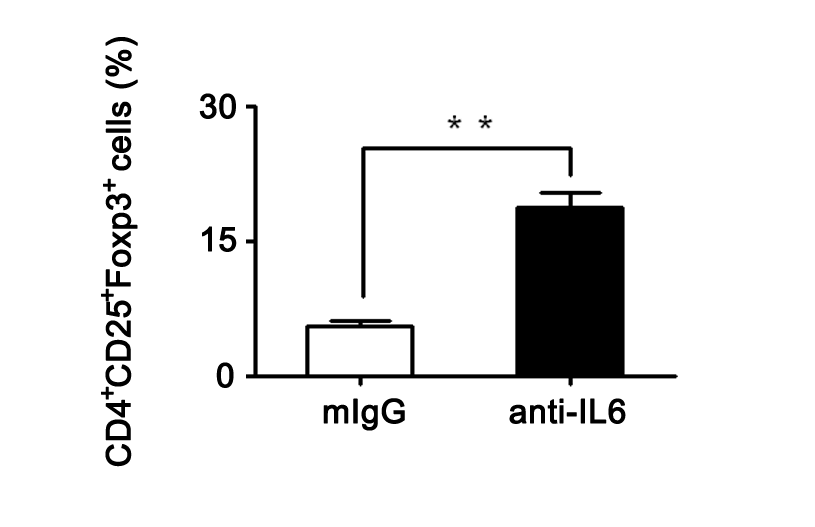

Supplement: Figure S5 — Inhibition of Treg differentiation was dependent on IL-6. Purified CD4+ T cells from naïve mice were cultured with mDCs in the presence of TGF-β and anti-IL-6 neutralizing antibody for 2 days, cells then were stained with anti-CD4, anti-CD25 and anti-Foxp3 Abs and analyzed on gated CD4+ T cells by FACS. Data are representatives of three independent experiments. **, p<0.01. (TIF) [file pone.0027289.s005.tif]
